# Visual Omics: a web-based platform for omics data analysis and visualization with rich graph-tuning capabilities

**DOI:** 10.1093/bioinformatics/btac777

**Published:** 2022-12-02

**Authors:** Heng Li, Mijuan Shi, Keyi Ren, Lei Zhang, Weidong Ye, Wanting Zhang, Yingyin Cheng, Xiao-Qin Xia

**Affiliations:** Institute of Hydrobiology, Chinese Academy of Sciences, Wuhan 430072, China; College of Advanced Agricultural Sciences, University of Chinese Academy of Sciences, Beijing 100049, China; Institute of Hydrobiology, Chinese Academy of Sciences, Wuhan 430072, China; The Innovative Academy of Seed Design, Chinese Academy of Sciences, Beijing 100101, China; College of Fisheries and Life Science, Dalian Ocean University, Dalian 116023, China; Institute of Hydrobiology, Chinese Academy of Sciences, Wuhan 430072, China; College of Advanced Agricultural Sciences, University of Chinese Academy of Sciences, Beijing 100049, China; Institute of Hydrobiology, Chinese Academy of Sciences, Wuhan 430072, China; College of Advanced Agricultural Sciences, University of Chinese Academy of Sciences, Beijing 100049, China; Institute of Hydrobiology, Chinese Academy of Sciences, Wuhan 430072, China; The Innovative Academy of Seed Design, Chinese Academy of Sciences, Beijing 100101, China; Institute of Hydrobiology, Chinese Academy of Sciences, Wuhan 430072, China; The Innovative Academy of Seed Design, Chinese Academy of Sciences, Beijing 100101, China; Institute of Hydrobiology, Chinese Academy of Sciences, Wuhan 430072, China; College of Advanced Agricultural Sciences, University of Chinese Academy of Sciences, Beijing 100049, China; The Innovative Academy of Seed Design, Chinese Academy of Sciences, Beijing 100101, China

## Abstract

**Summary:**

With the continuous development of high-throughput sequencing technology, bioinformatic analysis of omics data plays an increasingly important role in life science research. Many R packages are widely used for omics analysis, such as DESeq2, clusterProfiler and STRINGdb. And some online tools based on them have been developed to free bench scientists from programming with these R packages. However, the charts generated by these tools are usually in a fixed, non-editable format and often fail to clearly demonstrate the details the researchers intend to express. To address these issues, we have created Visual Omics, an online tool for omics data analysis and scientific chart editing. Visual Omics integrates multiple omics analyses which include differential expression analysis, enrichment analysis, protein domain prediction and protein–protein interaction analysis with extensive graph presentations. It can also independently plot and customize basic charts that are involved in omics analysis, such as various PCA/PCoA plots, bar plots, box plots, heat maps, set intersection diagrams, bubble charts and volcano plots. A distinguishing feature of Visual Omics is that it allows users to perform one-stop omics data analyses without programming, iteratively explore the form and layout of graphs online and fine-tune parameters to generate charts that meet publication requirements.

**Availability and implementation:**

Visual Omics can be used at http://bioinfo.ihb.ac.cn/visomics. Source code can be downloaded at http://bioinfo.ihb.ac.cn/software/visomics/visomics-1.1.tar.gz.

**Supplementary information:**

Supplementary data are available at *Bioinformatics* online.

## 1 Introduction

With the popularization of high-throughput sequencing technology, finding evidence from the perspective of omics has become a basic idea. Therefore, omics analysis has become an indispensable requirement for many laboratories. As a computer programming language specifically designed for statistical analysis, a large number of software packages for R language have been developed for processing high-throughput omics data, such as DESeq2 (Love *et al.*, 2014) for difference analysis, clusterProfiler (Wu *et al.*, 2021) for enrichment analysis, STRINGdb (Szklarczyk *et al.*, 2021) for protein interaction analysis, etc. Most of these R packages require users to have the ability to operate and program in R language, raising the threshold for ‘wet experiment’ researchers to use them to analyze data. In contrast, online tools such as DAVID (Huang *et al.*, 2009) and ImageGP (Chen *et al.*, 2022) do not have this problem, and users can directly upload, analyze and visualize data with these tools. NASQAR (Yousif *et al.*, 2020) even allows users interactively adjust the location of lines in diagrams. However, these online tools usually have limited graphical adjustment functions, which are not enough to comprehensively tune the various elements in a diagram. So, researchers often need to use other software to modify these standard pictures or plot new charts in order to express their assertions more accurately, or to meet the needs of scientific journals. In addition, because the color, grayscale, point size and other attributes in scientific figures are the mapping of real data, using image editing software to change these attributes later, such as scaling up the size of the point in figures, is not only cumbersome to operate, and it is easy to cause image distortion.

Here, we present Visual Omics, a one-stop web-based tool for omics data analysis and fine-tuning scientific figures. Visual Omics currently integrates differential expression analysis, enrichment analysis, protein domain prediction, protein interaction analysis and other omics analyses, as well as the independent and adjustable basic graphics drawing functions involved in omics analysis, including PCA/PCoA plots, bar plots, box plots, heat maps, UpSet plots, Venn diagrams, bubble charts and volcano plots. In addition to obtaining the corresponding analysis results, users can also perform iterative and high-degree of freedom parameter adjustment on important figures online until the newly generated figures meet personalized requirements.

## 2 Materials and methods

### 2.1 Omics information analysis

The omics data analysis functions of Visual Omics are mainly based on R (4.1.1) packages such as DESeq2 and clusterProfiler, and Perl (v5.32.1) tools such as pfam_scan.pl (Mistry *et al.*, 2021). (See Supplementary Table S1 for detailed function-dependent toolkits and Supplementary Table S2 for R packages and related references.)

For ease of use, Visual Omics provides brief help information for each analysis method at the top of each web page, including the workflow and usage of each analysis method, as well as example images of the main results. In addition, example files or sample data in input boxes are provided for users to learn the data format. After uploading data files or pasting the data in input boxes, the users can adjust the parameters of the analysis according to their needs. (See Supplementary Table S3 for the parameter statistics of each analysis process.) Clicking the ‘Submit’ button will trigger the backend program to perform calculations based on the input data, and return analysis results and graphs. If users want to make detailed adjustments to a graph, just click the ‘fine-tuning’ button below the corresponding graph to enter the online fine-tuning page. And most of the parameters of graphs are adjustable with visible effect. The initial graph and adjusted graphs are successively named ‘pic.1’, ‘pic.2’, …. Although only one graph is displayed in the graph area, users can switch to the *n*th graph by clicking its name ‘pic.n’ below the graph area and modify it through the ‘detail modify’ button. The graphs can be tweaked iteratively until a satisfactory one is obtained, after which the final graph and the analysis results can be downloaded. (See the ‘Fine tuning behavior’ section of the online Help page for details.)

### 2.2 Scientific graphics fine-tuning

**Fig. 1.**
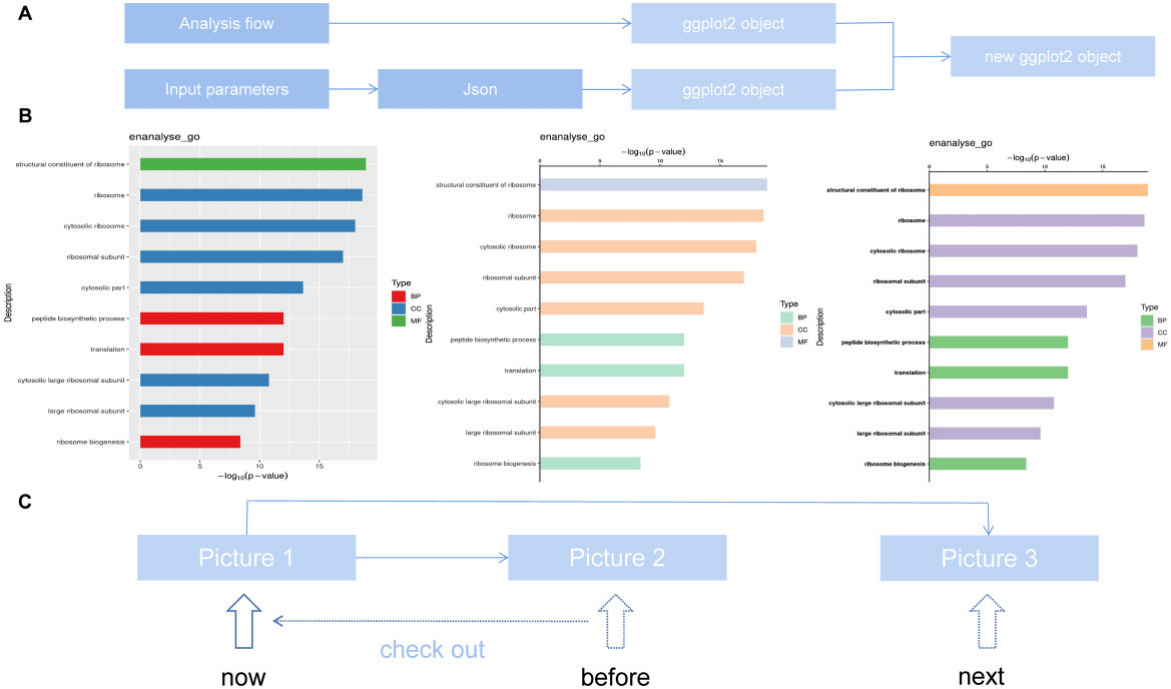
Principle of image adjustment. (**A**) Image adjustment is an additive modification of the original drawing object. (**B**) An example of an image modification of the bar chart of one of the GO enrichment analysis results from images and corresponds to the schematic diagram of C. (**C**) schematic diagram of working image-switching principle. Users can switch working images, modify the switching images and generate new working images

The online fine-tuning function of Visual Omics was implemented based on ggplot2 (Wickham, 2016) and its series R packages (see Supplementary Table S1 for details). In order to simulate the trial-and-error process of continuous modification and repeated backtracking when users actually retouch graphs, two characteristic working mechanisms were adopted in Visual Omics. One is for the additive modification of graphics layers. Visual Omics uses ggplot2 objects to visualize the analysis results as graphs, which are then adjusted by manipulating the ggplot2 objects. For a complete modification, user-entered parameters are organized into a JSON (https://www.ietf.org/rfc/rfc4627.txt) object and passed to the R script, which is then converted into a ggplot2 parameter object and used to update the original ggplot2 object (Fig. 1A). This mechanism directly realizes the additive modification of the graphs and also solves the problem of transferring a large number of graphics parameters. The other mechanism is for switching of working graphs. We designed a unique parameter transmission system that works similarly to Git, the widely used distributed version control system. With a single mouse click the user can easily switch to any version of the graphs that has been generated (Fig. 1C) and set the switched graph as the current working graph. All parameter modifications will be used to generate a new graph based on the working graph. With the additive modification and graphic switching capabilities, Visual Omics fully meets users' needs for continuous incremental exploration, fine-tuning, and freedom to backtrack on scientific graphs.

Since the user data will not be saved after the session, the user can download the ggplot2 object corresponding to any fine-tuned diagram and continue to adjust it in the future through the ‘Retuning Plots’ module of Visual Omics. More importantly, through the reset feature of the mapping function in ggplot2, Visual Omics can modify graphical properties such as the color ranges of heat maps and the point size range of a bubble charts or network diagram, without breaking the inherent connection between the data and the graph. This is something that desktop editing software cannot do.

## 3 Result and discussion

Visual Omics is deployed on a server equipped with 1 T running memory, 32 CPU cores and 200 T of storage space, a configuration that meets the typical data analysis needs. To avoid the tedious data uploads, data analysis and plotting in Visual Omics are based on downstream data in omics analysis, such as gene expression tables, rather than bulky raw sequence files. Given the privacy of user data, Visual Omics does not require users to provide background information about the data, without which the data can only be understood by the data owner. Furthermore, user-uploaded data will be stored in a folder with a random name of at least 76 characters and will be deleted immediately after the session.

Visual Omics integrates multiple omics analysis functions and plotting capabilities for the main types of graphs involved. The modification of graphs covers seven major parts such as coordinate axes, legends and data mapping (color, size and fill) (see the ‘Fine tuning parameters’ section of the Help page for details), and a total of 260 parameters can be used to fine tune 38 graphic elements (Supplementary Table S4), implying that images can be adjusted with a high degree of freedom (Table 1). With integrated bioinformatics tools and uniquely designed online graph adjustments, Visual Omics provides users with a one-stop service for transforming data into personalized and publication-grade results (Fig. 1B).

**Table 1.** Summary of analysis parameters and image adjustment parameters

Classification	Function	Parameters for analysis and initial plotting	Tuning parameters
Analysis process	Differential expression analysis	14	219–232
	Enrichment analysis	12	220–223
	Protein domain prediction	1	223
	Protein–protein interaction analysis	14	221
Base plots	Oridnations (PCA/PCoA plots)	8	232
	Bar plots	12	232
	Box plots	12	232
	Heat maps	11	219
	UpSet plots	13	–
	Venn diagrams	15	224
	Volcano plots	7	224
	Bubble charts	8	236
Retuning plots		8	219–236

As a tool focusing on the visualization of omics data, Visual Omics cannot compete with well-known omics analysis platforms such as Galaxy (Afgan *et al.*, 2018) and GenePattern (Reich *et al.*, 2006) in terms of the richness of data analysis functions. However, compared with some recent omics analysis tools such as NASQAR, OmincsNet 2.0 (Zhou *et al.*, 2022), PaintOmics (Tianyuan *et al.*, 2022), and even specialized omics visualization tools like esquisse (https://github.com/dreamRs/esquisse) and Gosling (L'Yi *et al.*, 2022), Visual Omics not only provides a more comprehensive ability to adjust graph elements but also has greater flexibility in adjustment exploration based on version control and cross-session retuning functions (Supplementary Table S5). The data processing flow required between the upstream omics analysis result and the standard input of the downstream analysis may also be integrated into Visual Omics in the future.

Visual Omics has a broad space for development, and its most potential is that it is an online implementation of R's ecological chain in omics data analysis, i.e. upstream data analysis and downstream visualization. This means that any upstream omics analysis pipeline implemented in R can be easily integrated into Visual Omics. The extensive use of the powerful downstream ggplot2 and its family packages enables almost all analysis results to be visualized by Visual Omics and can be adapted to the online tuning system almost without modification. With the deep accumulation and vigorous development trend of R in the field of omics analysis, Visual Omics will accommodate more omics analysis functions and further expands the range of adjustable elements in the image editing system, in order to allow wet experimenters without programming skills to be able to fully experience the integrated data analysis, visualization and online image adjustment with a high degree of freedom.

## Supplementary Material

btac777_Supplementary_Data

## References

[btac777-B1] Afgan E. et al (2018) The galaxy platform for accessible, reproducible and collaborative biomedical analyses: 2018 update. Nucleic Acids Res., 46, W537–W544. 29790989 10.1093/nar/gky379 PMC6030816

[btac777-B2] Chen T. et al (2022) ImageGP: an easy-to-use data visualization web server for scientific researchers. iMeta, 1, e5. 10.1002/imt2.5 PMC10989750 38867732

[btac777-B3] Huang D.W. et al (2009) Systematic and integrative analysis of large gene lists using DAVID bioinformatics resources. Nat. Protoc., 4, 44–57. 19131956 10.1038/nprot.2008.211

[btac777-B4] Mistry J. et al (2021) Pfam: the protein families database in 2021. Nucleic Acids Res., 49, D412–D419. 33125078 10.1093/nar/gkaa913 PMC7779014

[btac777-B5] Love M.I. et al (2014) Moderated estimation of fold change and dispersion for RNA-seq data with DESeq2. Genome Biol., 15, 550. 25516281 10.1186/s13059-014-0550-8 PMC4302049

[btac777-B6] L'Yi S. et al (2022) Gosling: a grammar-based toolkit for scalable and interactive genomics data visualization. IEEE Trans. Visual. Comput. Graphics, 28, 140–150. 10.1109/TVCG.2021.3114876 PMC8826597 34596551

[btac777-B7] Reich M. et al (2006) GenePattern 2.0. Nat. Genet., 38, 550–501. 16642009 10.1038/ng0506-500

[btac777-B8] Szklarczyk D. et al (2021) The STRING database in 2021: customizable protein-protein networks, and functional characterization of user-uploaded gene/measurement sets. Nucleic Acids Res., 49, D605–D612. 33237311 10.1093/nar/gkaa1074 PMC7779004

[btac777-B9] Tianyuan L. et al (2022) PaintOmics 4: new tools for the integrative analysis of multi-omics datasets supported by multiple pathway databases. Nucleic Acids Res., 50, W551–W559. 35609982 10.1093/nar/gkac352 PMC9252773

[btac777-B10] Wu T. et al (2021) clusterProfiler 4.0: a universal enrichment tool for interpreting omics data. Innovation (Cambridge), 2, 100141. 10.1016/j.xinn.2021.100141 PMC8454663 34557778

[btac777-B11] Wickham H. (2016) ggplot2: Elegant Graphics for Data Analysis. Springer, New York.

[btac777-B12] Yousif A. et al (2020) NASQAR: a web-based platform for high-throughput sequencing data analysis and visualization. BMC Bioinformatics, 21, 267. 32600310 10.1186/s12859-020-03577-4 PMC7322916

[btac777-B13] Zhou G. et al (2022) OmicsNet 2.0: a web-based platform for multi-omics integration and network visual analytics. Nucleic Acids Res., 49, W527–W533. 10.1093/nar/gkac376 PMC9252810 35639733

