# Supplementary figures and images for: Visual Omics: a web-based platform for omics data analysis and visualization with rich graph-tuning capabilities
Source: Bioinformatics. 2022 Dec 2;39(1):btac777. doi: 10.1093/bioinformatics/btac777 (PMC9825776; doi:10.1093/bioinformatics/btac777)

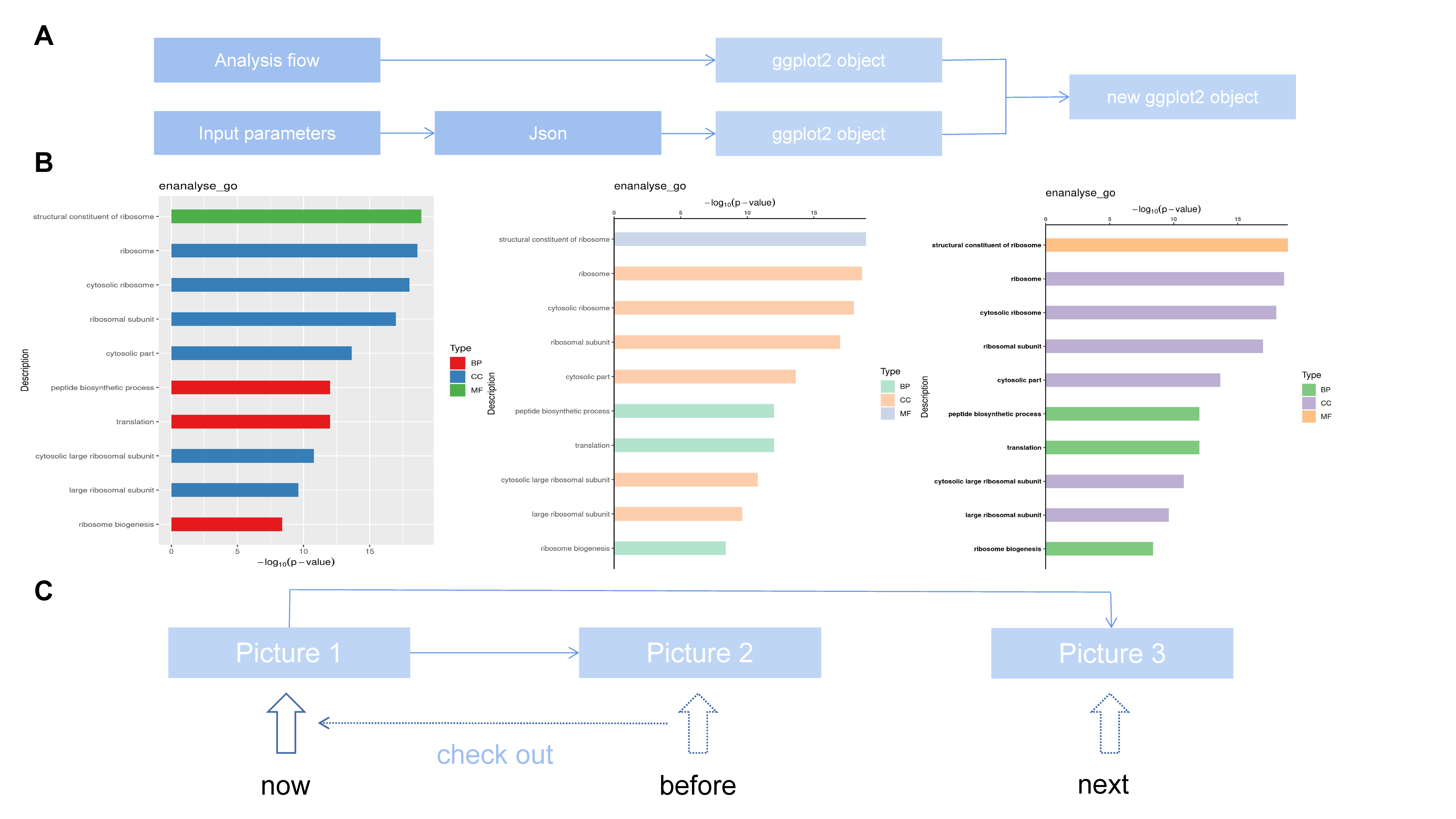

Supplement: btac777_Supplementary_Data [file btac777_supplementary_data.zip › Fig.1.tif]
